# Assisted Living Administrators’ Approaches to Advance Care Planning

**DOI:** 10.1093/geroni/igaa057.216

**Published:** 2020-12-16

**Authors:** Elise Abken, Alexis Bender, Ann Vandenberg, Candace Kemp, Molly Perkins

**Affiliations:** 1 Emory University School of Medicine, Atlanta, Georgia, United States; 2 Emory University, Atlanta, Georgia, United States; 3 Georgia State University, Atlanta, Georgia, United States

## Abstract

Assisted living (AL) communities are increasingly home to frail, chronically ill older adults who remain until death. State laws mandate that AL facilities request copies of any advance care planning documents residents have and make forms available upon request. Using secondary data from a larger study funded by the National Institute on Aging (R01AG047408) that focuses on end-of-life (EOL) care in AL, this project investigated barriers and facilitators to conducting advance care planning in AL. Data included in-depth interviews (of 86 minute average length) with 20 administrators from 7 facilities around the Atlanta metropolitan area and aggregate data collected from each facility regarding facility, staff, and resident characteristics. Findings from thematic analysis of qualitative data showed that key barriers to planning in AL included lack of staff training and reluctance among administrators and families to discuss advance care planning and EOL care. Important facilitators included periodic follow-up discussions of residents’ wishes, often during care plan meetings, educating families about the importance of planning, and external support for staff training and family education from agencies such as hospice and home health. Three study facilities exceeded state requirements to request and store documents by systematically encouraging residents to complete documentation. These facilities, whose administrators discuss advance care planning and residents’ EOL wishes with residents and families during regular care plan meetings, were more likely to have planning documents on file, demonstrating the potential of long-term care communities, such as AL, to successfully promote advance care planning among residents and their family members.

